# Supplementary material for: Complexome profiling of the Chlamydomonas psb28 mutant reveals TEF5 as an early PSII assembly factor
Source: Plant Cell. 2025 Mar 17;37(6):koaf055. doi: 10.1093/plcell/koaf055 (PMC12164590; doi:10.1093/plcell/koaf055)
Supplement: koaf055_Supplementary_Data [file koaf055_supplementary_data.zip › psb28_interactive.zip!/plots/intensity/qualityPlots/peptides/_Cre03.g162000.t1.2.html]

### Cre03.g162000.t1.2

Trivial name:   
SeqCoverage [median]: 6.90  
Mass: 37.98 kDa  
  
 

| Sequence | MissedCleavage | Mass [Da] | wtIntensity | muIntensity | Score |
| --- | --- | --- | --- | --- | --- |
| LQGARPPVPTTK | 1 | 1263.73 | 2410.70 | 6801.63 | 74.70 |
| SIPDLEKSPLLR | 1 | 1366.78 | 532.78 | 6249.68 | 64.55 |
|  |  |  | 2943.5 | 13051.3 |  |
